# Comparison of four automated microbiology systems with 16S rRNA gene sequencing for identification of *Chryseobacterium* and *Elizabethkingia* species

**DOI:** 10.1038/s41598-017-14244-9

**Published:** 2017-10-23

**Authors:** Jiun-Nong Lin, Chung-Hsu Lai, Chih-Hui Yang, Yi-Han Huang, Hsiu-Fang Lin, Hsi-Hsun Lin

**Affiliations:** 1 Department of Critical Care Medicine, E-Da Hospital, I-Shou University, Kaohsiung, Taiwan; 2 Division of Infectious Diseases, Department of Internal Medicine, E-Da Hospital, I-Shou University, Kaohsiung, Taiwan; 3 School of Medicine, College of Medicine, I-Shou University, Kaohsiung, Taiwan; 4 Department of Biological Science and Technology, Meiho University, Pingtung, Taiwan; 5 Departments of Clinical Pathology, E-Da Hospital, I-Shou University, Kaohsiung, Taiwan

## Abstract

*Chryseobacterium* and *Elizabethkingia* species have recently emerged as causative agents in life-threatening infections in humans. We aimed to evaluate the rates at which four common microbial identification systems identify *Chryseobacterium* and *Elizabethkingia* species in clinical microbiology laboratories. Based on the results of 16S rRNA gene sequencing, a total of 114 consecutive bacteremic isolates, including 36 (31.6%) *C*. *indologenes*, 35 (30.7%) *E*. *anophelis*, 22 (19.3%) *C*. *gleum*, 13 (11.4%) *E*. *meningoseptica*, and other species, were included in this study. The overall concordance between each method and 16S rRNA gene sequencing when identifying *Chryseobacterium* and *Elizabethkingia* species was 42.1% for API/ID32, 41.2% for Phoenix 100 ID/AST, 43.9% for VITEK 2, and 42.1% for VITEK MS. Among the 22 *C*. *gleum* isolates, only one (4.8%) was correctly identified using VITEK 2 and Phoenix 100 ID/AST, and none were accurately recognized using API/ID32 or VITEK MS. Except for two isolates that were not identified using API/ID32, all *E*. *anophelis* isolates were misidentified by all four identification systems as *E*. *meningoseptica*. Our results show that these approaches have low accuracy when identifying *Chryseobacterium* and *Elizabethkingia* species. Hence, we recommend amending the discrimination rate of and adding non-claimed pathogens to databases of microbial identification systems.

## Introduction

The genera *Chryseobacterium* and *Elizabethkingia* are aerobic, nonfermenting, nonmotile, catalase-positive, oxidase-positive, indole-positive, and gram-negative bacilli that are distributed in soil and water environments^1,2^. These microorganisms were recently been reported as agents that cause life-threatening pneumonia, bacteremia, meningitis, and neutropenic fever in humans, especially immunocompromised patients^1–5^.

Both *Chryseobacterium* and *Elizabethkingia* were historically derived from the genus *Flavobacterium*. To date, more than 90 species are included in the genus *Chryseobacterium*. Among these, *C*. *indologenes* is the most common cause of human infections^1,5^. Human infections caused by other *Chryseobacterium* species, such as *C*. *gleum*, are rarely reported^6^. Currently, the genus *Elizabethkingia* includes three species, including *E*. *meningoseptica*, *E*. *miricola*, and *E*. *anophelis* ^7^. Among these species, *E*. *meningoseptica* is the most well-known species that causes opportunistic infections in immunocompromised patients^2–4^. However, *Elizabethkingia anophelis* has recently emerged as a cause of life-threatening infections and has triggered several outbreaks of infections in Africa^8^, Singapore^9^, Hong Kong^10^, and the USA^11,12^.

Several methods have been developed to identify microorganisms. These include biochemical methods, 16S rRNA gene sequencing, multiple molecular marker sequencing, and protein fingerprinting techniques (for example, matrix-assisted laser desorption ionization–time of flight mass spectrometry; MALDI-TOF MS). Occasional studies have reported misidentifying *Chryseobacterium* and *Elizabethkingia* species when using conventional phenotypic identification systems and the VITEK 2 Automated Identification System (bioMérieux, Marcy l’Etoile, France)^6,10,13^. However, 16S rRNA gene sequencing has been shown to be a reliable method of identifying *Chryseobacterium* and *Elizabethkingia* species^14,15^. In this study, we used 16S rRNA sequencing to analyze *Chryseobacterium* and *Elizabethkingia* species isolated from patient blood samples. We compared the accuracies of the following four bacterial identification systems that are commonly used to identify *Chryseobacterium* and *Elizabethkingia* species: (1) API/ID32 Phenotyping Kits (bioMérieux, Marcy l’Etoile, France), (2) Phoenix 100 ID/AST Automated Microbiology System (Becton Dickinson Co., Sparks, MD, USA), (3) VITEK 2 Automated Identification System, and (4) VITEK MS MALDI-TOF MS System (bioMérieux, Marcy l’Etoile, France).

## Results

### Isolates and claimed microorganisms in databases of identification systems

A total of 114 consecutively non-repeated isolates that were initially identified as *Chryseobacterium* and *Elizabethkingia* species by a clinical microbiology laboratory were included in this study (Table 1). According to BLAST results based on 16S rRNA gene sequencing, 36 isolates were *C*. *indologenes* (31.6%), 35 were *E*. *anophelis* (30.7%), 22 were *C*. *gleum* (19.3%), 13 were *E*. *meningoseptica* (11.4%), 3 were *C*. *culicis*, 2 were *C*. *bemardetii*, 2 were *Candidatus Chryseobacterium massilia*, and 1 was *E*. *miricola*. The coverage rates of these *Chryseobacterium* and *Elizabethkingia* species that were obtained using the API/ID32, Phoenix 100 ID/AST, VITEK 2, and VITEK MS v2.0/v3.0 identification databases are shown in Table 1. *Chryseobacterium indologenes* and *E*. *meningoseptica* were claimed in the databases of all biochemical systems and MALDI-TOF MS systems. *C*. *gleum* was built into all systems except API/ID32. *Elizabethkingia miricola* was included only in the Phoenix 100 ID/AST database. However, these identification systems did not contain identification data for *Candidatus C*. *massilia*, *C*. *bemardetii*, *C*. *culicis*, and *E*. *anophelis*.

**Table 1:** The manufacturers’ listed coverage of databases and identification of *Chryseobacterium* and *Elizabethkingia* species by API/ID32, Phoenix 100 ID/AST, VITEK 2, and VITEK MS.

16S rRNA sequence-based identification (no. of isolates)	Identification method (no. of isolates)											
	API/ID32 v3.1			Phoenix 100 ID/AST v5.51A			VITEK 2 v7.01			VITEK MS Knowledge Base v2.0/v3.0		
	Strain name (no. of isolates)	Database Coverage	Correct rate (%)	Strain name (no. of isolates)	Database Coverage	Correct rate (%)	Strain name (no. of isolates)	Database Coverage	Correct rate (%)	Strain name (no. of isolates)	Database Coverage	Correct rate (%)
*Chryseobacterium* species (65)			55.4			53.8			56.9			53.8
*Candidatus C*. *massilia* (2)	*C*. *indologenes* (2)	No	0	*Sphingomonas paucimobilis* (2)	No	0	*C*. *indologenes* (1) Genus *Brevundimonas* (1)	No	0	*C*. *indologenes* (2)	No	0
*C*. *bemardetii* (2)	*C*. *indologenes* (2)	No	0	*C*. *indologenes* (1) *Bergeyella zoohelcum* (1)	No	0	*C*. *indologenes* (2)	No	0	Genus *Chryseobacterium* (1) No identification (1)	No	0
*C*. *culicis* (3)	*E*. *meningoseptica* (1) *C*. *indologenes* (2)	No	0	*C*. *indologenes* (2) *Bergeyella zoohelcum* (1)	No	0	*C*. *indologenes* (3)	No	0	*C*. *indologenes* (2) No identification (1)	No	0
*C*. *gleum* (22)	*C*. *indologenes* (18) Genus *Chryseobacterium* (1) No identification (3)	No	0	*C*. *gleum* (1) *C*. *indologenes* (14) *Bergeyella zoohelcum* (7)	Yes	4.5	*C*. *gleum* (1) *C*. *indologenes* (21)	Yes	4.5	*C*. *indologenes* (19) No identification (3)	Yes	0
*C*. *indologenes* (36)	*C*. *indologenes* (36)	Yes	100	*C*. *indologenes* (34) *Bergeyella zoohelcum* (2)	Yes	94.4	*C*. *indologenes* (36)	Yes	100	*C*. *indologenes* (35) No identification (1)	Yes	97.2
*Elizabethkingia* species (49)			24.5			24.5			26.5			26.5
*E*. *anopheles* (35)	*E*. *meningoseptica* (26) No identification (2)	No	0	*E*. *meningoseptica* (35)	No	0	*E*. *meningoseptica* (35)	No	0	*E*. *meningoseptica* (35)	No	0
*E*. *meningoseptica* (13)	*E*. *meningoseptica* (12) No identification (1)	Yes	92.3	*E*. *meningoseptica* (12) *Empedobacter brevis* (1)	Yes	92.3	*E*. *meningoseptica* (13)	Yes	100	*E*. *meningoseptica* (13)	Yes	100
*E*. *miricola* (*1*)	*E*. *meningoseptica* (1)	No	0	*E*. *meningoseptica* (1)	Yes	0	*E*. *meningoseptica* (1)	No	0	*E*. *meningoseptica* (1)	No	0
Quality control strain												
*C*. *indologenes* BCRC 17271	*C*. *indologenes*	Yes	100	*C*. *indologenes*	Yes	100	*C*. *indologenes*	Yes	100	*C*. *indologenes*	Yes	100
*E*. *meningoseptica* BCRC 10677	*E*. *meningoseptica*	Yes	100	*E*. *meningoseptica*	Yes	100	*E*. *meningoseptica*	Yes	100	*E*. *meningoseptica*	Yes	100

### API/ID32 Phenotyping Kits

The API/ID32 system correctly identified all isolates of *C*. *indologenes* (36/36) and 92.3% (12/13) of *E*. *meningoseptica* isolates (Table 1). Eighteen of 22 *C*. *gleum* (81.8%) were misidentified as *C*. *indologenes*. Six of 7 rarely observed *Chryseobacterium* (including *Candidatus C*. *massilia*, *C*. *bemardetii*, and *C*. *culicis*) were recognized as *C*. *indologenes*, and the seventh was identified as *E*. *meningoseptica*. The majority (94.4%) of *E*. *anophelis* and *E*. *miricola* were identified as *E*. *meningoseptica*. The overall correct rates of identification of *Chryseobacterium* and *Elizabethkingia* species when using API/ID32 were 55.4% and 24.5%, respectively.

### Phoenix 100 ID/AST Automated Microbiology System

Among the 36 isolates of *C*. *indologenes*, 34 (94.4%) were correctly identified. However, only 4.5% (1/22) of *C*. *gleum* were successfully diagnosed. No *Candidatus C*. *massilia*, *C*. *bemardetii*, and *C*. *culicis* were recognized (Table 1). The accuracy rate when identifying *Chryseobacterium* species was 53.8%. For the genus *Elizabethkingia*, 92.3% (12/13) of *E*. *meningoseptica* were accurately identified. However, the other two species, *E*. *anophelis* and *E*. *miricola*, were misdiagnosed as *E*. *meningoseptica*. The rate of successful identification of *Elizabethkingia* species was only 24.5% when using the Phoenix 100 ID/AST Automated Microbiology System.

### VITEK 2 Automated Identification System

All *C*. *indologenes* (n = 36) were reliably identified when using VITEK 2 (Table 1). However, 4.5% (1/22) of *C*. *gleum* species were correctly identified, and the remaining 95.5% (21/22) were misidentified as *C*. *indologenes*. All *C*. *bemardetii* and *C*. *culicis* isolates were identified as *C*. *indologenes*. Of the *Elizabethkingia* species, all *E*. *meningoseptica* (13/13) were successfully identified, but the remaining three species were misidentified as *E*. *meningoseptica*. The accuracy of this method for identifying *Chryseobacterium* and *Elizabethkingia* species was 56.9% and 26.5%, respectively.

### VITEK MS MALDI-TOF MS System

Of the *Chryseobacterium* species, 97.2% (35/36) of *C*. *indologenes* isolates were correctly identified. However, no *Candidatus C*. *massilia*, *C*. *bemardetii*, or *C*. *culicis* isolates were successfully recognized (Table 1). The overall rate at which this method correctly identified *Chryseobacterium* species was 53.8%. Of the *Elizabethkingia* species, all *E*. *meningoseptica* (13/13, 100%) were correctly identified. However, all *E*. *anophelis* and *E*. *miricola* were misidentified as *E*. *meningoseptica*. The accuracy rate of this method when identifying *Elizabethkingia* species was only 26.5%.

## Discussion

In this study, we compared the accuracies of four commercial microbial identification systems to that of 16S rRNA gene sequencing for identifying *Chryseobacterium* and *Elizabethkingia* species. The overall concordance between each of these four commercial methods and 16S rRNA gene sequencing for identifying *Chryseobacterium* and *Elizabethkingia* species were as follows: API/ID32, 42.1%; Phoenix 100 ID/AST, 41.2%; VITEK 2, 43.9%; and VITEK MS, 42.1%. After taking the coverage of each database into account, the overall concordance between 16S rRNA gene sequencing and API/ID32, Phoenix 100 ID/AST, VITEK 2, and VITEK MS was 98%, 75.8%, 70.4%, and 67.6%, respectively.

*Chryseobacterium gleum* is rarely reported to cause infection in humans^16,17^. However, our data reveal that *C*. *gleum* accounts for 33.8% of *Chryseobacterium* bacteremia cases in humans. Lo *et al*.^6^ reported that 15 clinical isolates of *C*. *gleum* that were confirmed by 16S rRNA gene sequencing were misidentified by VITEK 2 as *C*. *indologenes* (14/15; 93.3%) and *E*. *meningoseptica* (1/15; 6.7%). When submitted to a Bruker Microflex LT MALDI-TOF MS System using Biotyper database 3.0 (Bruker Daltonics, Bremen, Germany), 2 (13.3%) and 13 (86.6%) of these 15 isolates were identified as *C*. *gleum* species and probable species, respectively. In our study, 81.8% of *C*. *gleum* isolates were misidentified as *C*. *indologenes* by all four commercial identification systems. *Chryseobacterium gleum* was included in the Phoenix 100 ID/AST, VITEK 2, and VITEK MS (v2.0 and v3.0) databases but not in the API/ID32 database. Among the 22 *C*. *gleum* isolates in our study, only 1 (4.5%) was correctly identified by VITEK 2 and Phoenix 100 ID/AST, and none were accurately recognized by VITEK MS. MALDI-TOF MS systems have become popular in clinical microbiology laboratories because they rapidly, highly accurately, and cost-effectively identify different microorganisms. However, despite the fact that *C*. *gleum* was included in the spectral database, none of the *C*. *gleum* isolates were accurately identified by VITEK MS. The four microbial identification systems used in our study are widely used by clinical microbiology laboratories all over the world. The inability of these techniques to distinguish *C*. *gleum* from *C*. *indologenes* may result from false impressions that have led to the notion that there is a low prevalence of *C*. *gleum* and an overestimation of the prevalence of *C*. *indologenes* infections in humans.

Recent studies have shown that *E*. *anophelis* is frequently misidentified as *E*. *meningoseptica* ^8–10^. Lau *et al*.^10^ reported 17 patients in Hong Kong who were diagnosed using 16S rRNA gene sequencing with infection with *E*. *anophelis*. However, all 17 *E*. *anophelis* isolates were recognized as *E*. *meningoseptica* by VITEK 2, and the Bruker MALDI-TOF MS Biotyper also failed to correctly identify *E*. *anophelis* ^10^. Similar to a report by Lau *et al*., Han *et al*.^13^ found in their study performed in South Korea that none of the tested 51 *E*. *anophelis* isolates was correctly identified by a Bruker MALDI-TOF MS Biotyper. A VITEK MS research-use-only (RUO) system coupled with a SARAMIS SuperSpectra database successfully identified all 51 *E*. *anophelis* isolates^13^, but this system is not available to clinical microbiology laboratories. In our study, 55.5% (27/49) of previously identified *E*. *meningoseptica* were revealed to be *E*. *anophelis* based on the results of 16S rRNA gene sequencing. Our results show that using VITEK MS with the v2.0 and v3.0 Knowledge Bases or any of the other three commonly used biochemical systems discussed here resulted in the failed identification of *E*. *anophelis*. We suggest that many previously reported *E*. *meningoseptica* infections might in fact have been identified as *E*. *anophelis* if they were analyzed using commercial identification systems. The prevalence of *E*. *anophelis* infections in humans could therefore be dramatically underestimated.

## Conclusions

Being able to correctly identify microorganisms is extremely important in clinical practice and microbiologic research. However, the results of our study show that four microbial identification systems that are widely used in clinical microbiology laboratories are highly inaccurate when identifying *Chryseobacterium* and *Elizabethkingia* species. Specifically, the extremely low rates at which these methods identify the life-threatening pathogens *C*. *gleum* and *E*. *anophelis* may cause the prevalence of these species to be substantially underestimated. We recommend amending the method used to discriminate *C*. *gleum* from *C*. *indologenes* and adding a database for *E*. *anophelis* to the microbial identification systems discussed here.

## Materials and Methods

### Ethics and experimental biosafety statements

This study was approved by the Institutional Review Board of E-Da Hospital (EMRP-105–134). The need for patient informed consent was waived by the Institutional Review Board of E-Da Hospital because the retrospective analysis of routine blood cultures posed no more than a minimal risk of harm to the subjects. The experiments in this study were approved by the Institutional Biosafety Committee of E-Da Hospital. All experiments were performed in accordance with relevant guidelines and regulations.

### Study design

An 11-year retrospective study was conducted at a 1,000-bed university-affiliated hospital that serves more than 2 million people in southern Taiwan. A clinical laboratory database was searched to identify blood cultures that were identified as containing *Chryseobacterium* and *Elizabethkingia* species between January 2005 and December 2015. The isolates were initially identified as *Chryseobacterium* and *Elizabethkingia* species by a clinical microbiology laboratory that first used API/ID32 Phenotyping Kits (2005–2013) and then used a VITEK MS MALDI-TOF MS System (2014–2015) after upgrading the microbial identification system. All isolates were stored as glycerol stocks at −80 °C until used. *Chryseobacterium indologenes* BCRC 17271 (ATCC 29897) and *Elizabethkingia meningoseptica* BCRC 10677 (ATCC 13253) were used as quality controls. The 16S rRNA gene sequencing method was considered the reference method for bacterial identification.

### 16S rRNA gene sequencing

Frozen bacterial glycerol stocks were thawed and subcultured on tryptic soy agar with 5% sheep blood (Becton Dickinson Co., Sparks, MD, USA) for further experiments. Total DNA was isolated from each sample using a Wizard Genomic DNA Purification Kit according to the manufacturer’s instructions (Promega, Madison, WI, USA). The primers used to amplify the internal fragments of the 16S rRNA gene were as described previously^18^. Purified polymerase chain reaction (PCR) was performed using a GeneAmp PCR System 9700 (Applied Biosystems, Foster City, CA, USA). The PCR products were sequenced using an Applied Biosystems 3730xl DNA Analyzer (Applied Biosystems, Foster City, CA, USA). The primers used to sequence 16S16S rRNA were 8f (5′-GGATCCAGACTTTGATYMTGGCTCAG-3′), 534r (5′-ATTACCGCGGCTGCTGG-3′), 534f (5′-CCAGCAGCCGCGGTAAT-3′), 968f (5′-AACGCGAAGAACCTTAC-3′), and 1512r (5′-GTGAAGCTTACGGYTAGCTTGTTACGACTT-3′)^19^. The sequences were reviewed and edited using Sequence Scanner v.1.0 (Applied Biosystems, Foster City, CA, USA). The obtained 16S rRNA sequences were compared to sequences in GenBank using the Basic Local Alignment Search Tool (https://blast.ncbi.nlm.nih.gov/Blast.cgi). The results were considered valid if the homologous rate was ≥99%.

### Identification of microorganisms using microbial identification systems

For re-identification, the thawed bacteria were inoculated on tryptic soy agar with 5% sheep blood after they were removed from the freezer. The plates were then incubated in a 5% CO_2_ atmosphere at 35 °C for 15 to 24 hours. All isolates were re-identified using API/ID32 Phenotyping Kits, Phoenix 100 ID/AST Automated Microbiology System, VITEK 2 Automated Identification System, and VITEK MS MALDI-TOF MS System. The isolates were identified according to each manufacturer’s instructions. For the API/ID32 Phenotyping Kits, an ID 32 GN card and database version 3.1 were used to identify microorganisms according to ATB Expression. The results obtained using the Phoenix 100 ID/AST System were analyzed using database version 5.51 A. A confidence level of ≥90% was defined as acceptable for the Phoenix System^20^. The identifications yielded by the VITEK 2 system were obtained using a GN ID card and database version 7.01. The quality of bacterial identification was assessed using VITEK 2 Advanced Expert System. The results were defined as acceptable at a confidence level of 96–99% (excellent identification) or 93–95% (very good quality)^21^. The mass spectral fingerprints generated by the VITEK MS System were analyzed using Knowledge Base v2.0 and repeatedly tested using Knowledge Base v3.0. A confidence value of ≥90% (reliable identification) or 85%–90% (acceptable identification) was regarded as a successful identification. A value was defined as no identification if the VITEK MS confidence value was <85%^22^.

### Data Availability

The datasets generated and analyzed during the current study are available from the corresponding author on reasonable request.

## References

[1] Chen FL (2013). Clinical and epidemiological features of *Chryseobacterium indologenes* infections: analysis of 215 cases. J. Microbiol. Immunol. Infect..

[2] Jean SS, Lee WS, Chen FL, Ou TY, Hsueh PR (2014). *Elizabethkingia meningoseptica*: an important emerging pathogen causing healthcare-associated infections. J. Hosp. Infect..

[3] Lin PY (2004). Clinical and microbiological analysis of bloodstream infections caused by *Chryseobacterium meningosepticum* in nonneonatal patients. J. Clin. Microbiol..

[4] Hung PP, Lin YH, Lin CF, Liu MF, Shi ZY (2008). *Chryseobacterium meningosepticum* infection: antibiotic susceptibility and risk factors for mortality. J. Microbiol. Immunol. Infect..

[5] Lin YT (2010). Clinical and microbiological characteristics of *Chryseobacterium indologenes* bacteremia. J. Microbiol. Immunol. Infect..

[6] Lo HH, Chang S-M (2014). Identification, characterization, and biofilm formation of clinical *Chryseobacterium gleum* isolates. Diagn. Microbiol. Infect. Dis..

[7] Nicholson, A. C., Humrighouse, B. W., Graziano, J. C., Emery, B. & McQuiston, J. R. Draft genome sequences of strains representing each of the *Elizabethkingia* genomospecies previously determined by DNA-DNA hybridization. *Genome Announc*. 4 (2016). 10.1128/genomeA.00045-16 PMC4786648 26966213

[8] Frank T (2013). First case of *Elizabethkingia anophelis* meningitis in the Central African Republic. Lancet.

[9] Teo J (2013). First case of *E anophelis* outbreak in an intensive-care unit. Lancet.

[10] Lau SKP (2016). *Elizabethkingia anophelis* bacteremia is associated with clinically significant infections and high mortality. Sci. Rep..

[11] CDC. Recent Outbreaks, *Elizabethkingia*. https://www.cdc.gov/elizabethkingia/outbreaks/ (2016).

[12] Navon L (2016). Notes from the Field: Investigation of *Elizabethkingia anophelis* Cluster - Illinois, 2014-2016. MMWR..

[13] Han MS (2017). Relative prevalence and antimicrobial susceptibility of clinical isolates of *Elizabethkingia* species based on 16S rRNA gene sequencing. J. Clin. Microbiol..

[14] Janda JM, Abbott SL (2007). 16S rRNA gene sequencing for bacterial identification in the diagnostic laboratory: pluses, perils, and pitfalls. J. Clin. Microbiol..

[15] Holmes B, Steigerwalt AG, Nicholson AC (2013). DNA-DNA hybridization study of strains of *Chryseobacterium*, *Elizabethkingia* and *Empedobacter* and of other usually indole-producing non-fermenters of CDC groups IIc, IIe, IIh and IIi, mostly from human clinical sources, and proposals of *Chryseobacterium bernardetii* sp. nov., *Chryseobacterium carnis* sp. nov., *Chryseobacterium lactis* sp. nov., *Chryseobacterium nakagawai* sp. nov. and *Chryseobacterium taklimakanense* comb. nov. Int. J. Syst. Evol. Microbiol..

[16] Kirby JT, Sader HS, Walsh TR, Jones RN (2004). Antimicrobial susceptibility and epidemiology of a worldwide collection of *Chryseobacterium* spp: report from the SENTRY Antimicrobial Surveillance Program (1997-2001). J. Clin. Microbiol..

[17] Arouna O (2017). *Chryseobacterium gleum* in a man with prostatectomy in Senegal: a case report and review of the literature. J. Med. Case Reports.

[18] Felske A, Rheims H, Wolterink A, Stackebrandt E, Akkermans AD (1997). Ribosome analysis reveals prominent activity of an uncultured member of the class *Actinobacteria in grassland so*ils. Microbiol..

[19] Hantsis-Zacharov E, Shakéd T, Senderovich Y, Halpern M (2008). *Chryseobacterium oranimense* sp. nov., a psychrotolerant, proteolytic and lipolytic bacterium isolated from raw cow’s milk. Int. J. Syst. Evol. Microbiol..

[20] O’Hara CM (2006). Evaluation of the Phoenix 100 ID/AST system and NID panel for identification of *Enterobacteriaceae*, *Vibrionaceae*, and commonly isolated nonenteric gram-negative bacilli. J. Clin. Microbiol..

[21] Schröttner P, Rudolph WW, Eing BR, Bertram S, Gunzer F (2014). Comparison of VITEK2, MALDI-TOF MS, and 16S rDNA sequencing for identification of *Myroides odoratus* and *Myroides odoratimimus*. Diagn. Microbiol. Infect. Dis..

[22] Jamal W, Albert MJ, Rotimi VO (2014). Real-time comparative evaluation of bioMerieux VITEK MS versus Bruker Microflex MS, two matrix-assisted laser desorption-ionization time-of-flight mass spectrometry systems, for identification of clinically significant bacteria. BMC Microbiol..

